# The SIAMS-ED Trial: A National, Independent, Multicentre Study on Cardiometabolic and Hormonal Impairment of Men with Erectile Dysfunction Treated with Vardenafil

**DOI:** 10.1155/2014/858715

**Published:** 2014-05-15

**Authors:** Andrea M. Isidori, Giovanni Corona, Antonio Aversa, Daniele Gianfrilli, Emmanuele A. Jannini, Carlo Foresta, Mario Maggi, Andrea Lenzi, SIAMS-ED Study Group, Sebastiano Andò, Gabriella Angelletti, Baldi Matteo, Giancarlo Balercia, Bellastella Giuseppe, Aldo E. Calogero, Domenico Canale, Massimiliano Caprio, Nicola Caretta, Ciotoli Erennio, Giovanni Maria Colpi, Andrea Fabbri, Riccardo Fornengo, Sandro Francavilla, Davide Francomano, Silvia Gavioli, Giagulli Vito Angelo, Elisa Giannetta, Umberto Goglia, Ilacqua Nicola, Sandro La Vignera, Andrea Lemma, Mario Mancini, Chiara Manieri, Riccardo Mansani, Ministrini Tommaso, Francesco Minuto, Oppo Alessandro, Francesca Paggi, Pivonello Rosario, Pelliccione Fiore, Perri Anna, Sebastio Perrini, Pofi Riccardo, Carlotta Pozza, Emilia Sbardella, Serra Stefano, Antonio Sinisi

**Affiliations:** ^1^Department Experimental Medicine, Sapienza University, 00161 Rome, Italy; ^2^Endocrinology Unit, Maggiore-Bellaria Hospital, 40133 Bologna, Italy; ^3^University of Tor Vergata, Department of System Medicine, 00133 Rome, Italy; ^4^Centre Cryopreservation of Male Gamete, University of Padova, 35122 Padua, Italy; ^5^Biomedicine, University of Florence, 50121 Florence, Italy; ^6^SIAMS, Italian Society of Andrology and Sexual Medicine, Italy

## Abstract

Increased cardiovascular risk has been associated with reduced response to proerectile drugs. The Italian Society of Andrology and Sexual Medicine (SIAMS) promoted an independent, multicenter study performed in 604 men (55 ± 12 yrs) suffering from erectile dysfunction (ED) to assess multiple health outcomes and response to 6-month vardenafil challenge in a real-life setting. Overall, 30.8% men had metabolic syndrome. Cardiovascular risk stratification revealed a greater number of ED subjects with moderate risk of a major adverse cardiovascular event than the general population (*P* < 0.01). Age-adjusted pulse pressure was positively correlated with ED severity and negatively with androgens and waist circumference (*P* < 0.01). A decline in total testosterone was observed with increasing arterial pulse pressure (*P* < 0.05), which was not accompanied by compensatory LH rise. Follow-up on 185 men treated with vardenafil in an nonrandomized, open, single-arm trial documented a significant rise in IIEF-5 (delta = 6.1 ± 4.8) that was maintained in men with high cardiovascular risk. Mild adverse events occurred in <5%, with no differences between cardiovascular risk classes. In summary, ED is a frequent symptom in patients with an elevated, but often unknown, risk of future cardiovascular events. Androgens predict vascular resistance in ED patients. Vardenafil's response and safety profile were preserved in subjects with higher cardiovascular risk.

## 1. Introduction

Large discrepancies have been reported for the prevalence of ED in the general population, and CV risk accounts for a significant part of this variability. Over the age of 40 years, moderate to severe ED is reported in about 30 to 35% of men [1]. However in men with hypertension the prevalence ranges from 26% to 68% and in those matching the diagnostic criteria for metabolic syndrome (MS) it ranges from 34% to 90% [2–4], with a progressive impairment of erectile function (EF) in parallel with the number of metabolic syndrome criteria met [5]. Since scientific societies revisited the diagnostic criteria for metabolic syndrome, there have been a number of studies describing its association with ED. Despite a clear pathophysiological link, it is still under debate whether the diagnosis of metabolic syndrome offers any diagnostic advantage, to the validated CV risk charts, for management of ED in real-life settings [6–8].

There is an unmet need of good CV risk biomarkers for ED subjects [9]. Recent epidemiological studies showed that reduced testosterone (TT) levels in men are associated with high blood pressure (BP), left ventricular mass, and increased cardiovascular mortality [10]. This may be due to the stiffening of large arteries, as observed in men under androgen ablation therapy for prostate cancer [11]. Pulse pressure (PP), the arithmetic difference between systolic (SBP) and diastolic blood pressure (DBP) [12, 13], has been considered to reflect arterial stiffness, an independent CV risk factor for elderly and diabetic patients [14–17]. We have previously reported an association between PP and arteriogenic ED [18], suggesting a negative association between penile blood flow and PP even after adjustment for mean BP and other confounders such as metabolic syndrome. Nevertheless, despite encouraging preliminary data, the relationship between testosterone levels, hypertension, and ED is far from being clarified [19, 20].

In Europe, systematic coronary risk evaluation (SCORE) charts have been validated for CVR. However, charts not developed in the country of application can lead to inadequate risk stratification [21]. The Italian* Progetto Cuore*  (Heart Project) has been extensively tested and shows an excellent overlap with the Mediterranean populations derived from the European SCORE [22]. The performance of such defined CV risk classes in predicting the severity of ED has yet to be documented.

Finally, men with several CVRFs are also less likely to benefit from standard ED treatments [23, 24]. Sponsored clinical trials for drug registration purposes are often biased by the tendency to recruit populations with lower CVRs than seen in end users in a real-life setting [25]. Therefore, the safety and efficacy of PDE5i in high CV risk populations require further investigation.

For all these reasons the Italian Society of Andrology and Sexual Medicine promoted a nationwide, independent, multicentre, two-phase study, consisting in a large cross-sectional analysis followed by an interventional, nonrandomized, open-label, single-arm trial carried out in the real-life settings. Its aim was to address all the following clinical questions. (1) What is the prevalence of cardiovascular and metabolic risk factors in unselected men attending an outpatient clinic for ED? (2) Does the diagnostic criteria of metabolic syndrome add any significant contribution to the CV risk chart for ED stratification? (3) What are the best determinants of the severity of ED in real-life settings? (4) Is PP an independent contributor to ED? (5) Does PP depend on androgen levels? (6) Is vardenafil's response and safety preserved in subjects with higher CV risk?

## 2. Methods

### 2.1. Study Protocol

**Figure 1:**
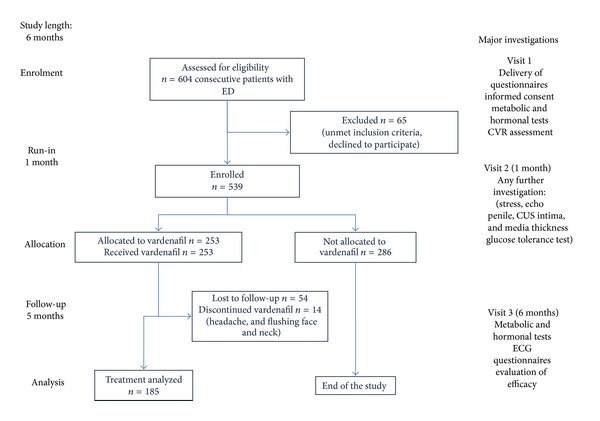
Study design (STARD plot).

In this independent, multicentre, open-label, prospective, noncomparative, interventional study, 604 consecutive men with ED (>6-month duration), as assessed by the international index of erectile function-5 (IIEF-5) [26], were recruited (Figure 1). The study was conducted from 2009 to 2011 at 18 public andrology and sexual medicine centres in Italy (see contributors). The protocol consisted of screening visit (V1), a 4-week washout period from any ED treatment (V2) and a 5/6-month treatment with vardenafil (any dose), prescribed at the investigator's discretion, and final response evaluation visit (V3). The interventional study was non-randomized and single blind. Subjects taking steroid hormones and other drugs known to affect directly testosterone levels were excluded from the study (Figure 1).

The entire study, from recruitment of centres to completion of the patient case report form (CRF), was carried out using web-based applications. The trial was registered on the Italian Society of Andrology and Sexual Medicine website (http://www.siams.info/) in 2008. Participation was open to any Italian NHS andrology outpatient clinic with proven experience in conducting ED trials. Interested sites could apply online, and eligible sites received the protocol kit by e-mail. In two start-up meetings, a training session was organized to guarantee uniformity in ultrasound techniques and diagnostic criteria. Monitoring of CRF completion, statistical analysis, and quality control was centralized at the Department of Experimental Medicine, Sapienza University of Rome. The protocol was approved by the Policlinico Umberto I Ethics Committee (Authorization 304/09) and by each local participating centre. All patients were asked to sign an informed consent form.

General details, medical and surgical history, concomitant medication history, and CV risk score—evaluated using the PC based risk engine derived from the* Progetto Cuore* study—were recorded at V1 [27]. The* Progetto Cuore*, launched in the late 1990s by the Italian Ministry of Health and based on the European systematic coronary risk evaluation (SCORE) risk charts, uses a computerized engine to record and follow up cardiovascular mortality and morbidity in the adult Italian population [21]. It allows us to collect data from Italian GPs that have downloaded the software for the stratification of CVR. The algorithm is used by a large number of GPs that are entering, remotely, the risk factors of their patients to provide an updated national estimate of the CV risk in the general Italian population. Epidemiological data on CV risk of the population are periodically released, enabling comparison of subgroups of interest (the latest release can be found at http://www.cuore.iss.it/). The database can be queried to generate the reference dataset of subgroup of interest; for the present study we used estimates from all males who were entered in the engine and matched for age, in the same time frame, with no other restriction criteria.

The following data were collected at baseline and after 6 months: general physical examination, weight, height, body mass index (BMI), waist circumference (WC), systolic blood pressure and DBP, PP calculated as the difference between systolic blood pressure and DBP, heart rate (HR), and the IIEF-5 questionnaires.

An IIEF-5 score of 21 or less confirmed ED, whose severity was classified into 4 categories: mild [17–21], mild to moderate [12–16], moderate [8–11], and severe [<8]. These categories were also merged to enable comparison of the first three (mild to moderate) against the last (severe). A clinical score based on a physician-guided interview was also analysed, as previously described [28]. Metabolic syndrome was defined according to the National Cholesterol Education Program's Adult Treatment Panel III (NCEP-ATP-III) [29]. Hormonal assessment included TT, luteinizing hormone (LH), and follicle stimulating hormone (FSH) [30]. Testosterone was measured by electrochemiluminescence (method: Immulite 2000 Siemens, Milan, Italy; within and between-assay coefficients of variation were 5.1% and 7.2%), while FSH and LH were measured using direct chemiluminescence (ADVIA Centaur, Bayer Co, Germany). A penile colour doppler ultrasound (PDCU) was also evaluated for all treated patients after a challenge with vasoactive agents according to previously published procedures [31]. Efficacy of vardenafil treatment was evaluated as variation from baseline in IIEF-5 scores (Δ-IIEF-5).

### 2.2. Response to Treatment in High CV Risk Subjects

Recruited subjects were stratified according to CV risk class as high (≥III, i.e., ≥10% risk of major cardiovascular event, [MACE]) versus low risk (<III, i.e., <10%). The primary efficacy variable was the variation in the IIEF-5 score at week 22–26 or last observation carried forward (LOCF) compared to baseline (Δ-IIEF-5). Secondary efficacy variables included the percentage of subjects achieving a “return-to-normal” erectile function (IIEF-5 > 22) and change in ED severity class. Safety variables included adverse events (AEs) recorded at all visits after visit 2 and vital signs, supine, and standing heart rate and BP.

Efficacy was analysed in both the intent-to-treat (ITT) population (patients who had taken at least one dose of study medication and had a LOCF) and the per-protocol (PP) population (patients who satisfied the ITT and had completed the 22 weeks of treatment). The safety population included all subjects taking at least one dose of vardenafil and having at least one reported postbaseline measurement. The incidence of treatment-emergent adverse events was analysed and reported to the central EC.

### 2.3. Statistical Analysis

The analyses were performed by ITT and PP set strategies, using SPSS 19.0 (SPSS Inc., Chicago, IL, USA). An alpha value threshold of 0.05 was used. The sample size was estimated to achieve a >80% power for rejection of the null hypothesis that a greater than 20% difference in the baseline-to-endpoint improvement in erectile function measured by IIEF-5 (Δ-IIEF-5) would occur between low and high CV risk classes. The expected ratio of low versus high CVR classes was estimated on the basis of the distribution in the general population (3 : 1). Sample size was therefore defined on the basis of the smaller group (high risk). Significance at the 0.05 level was required to reject the null hypothesis. All statistical tests were two tailed. Continuous variables were tested for normality (Shapiro-Wilk test); normal data were presented as mean ± SD, or as median with 25th–75th percentiles, if not otherwise specified. Comparisons were performed, according to their distribution, using Student's *t*-test for paired and unpaired data or Pearson's *χ* ^2^ test and Mann-Whitney Wilcoxon's signed-rank test. Associations among variables were tested with Spearman's correlation analysis. Linear regression analysis was used to explore determinants of total IIEF-5 score including, in a stepwise approach, all variables with a significant univariate association with measures of EF. Logistic regression analysis was used to identify predictors of severe ED. All analyses were performed adjusting for the effect of centre (18 recruiting sites). Changes in IIEF-5 score before and after treatment were analysed with a repeated measures analysis of variance model adjusted for confounding factors (ANCOVA). The same model was used to test whether response to treatment differed among groups with different CV risks.

## 3. Results

### 3.1. Characteristics of the Study Population

Six hundred and four men (median age of 55 years) with ED were assessed for eligibility, and 539 (89.2%) were enrolled in and completed the observational study. After completion, 253 spontaneously continued into the interventional study and were prescribed vardenafil on demand (10/20 mg, recommended twice weekly) for 5 consecutive months; 183 (73%) completed the interventional trial (Figure 1). The study population was representative of a real-life outpatient's clinic (Table 1): 17.8% had severe ED, 21.5% had moderate, 37.7% had mild-to-moderate, and 22.9% had mild ED. A large number of them were receiving treatments for underlying conditions: 253 (42%) of men were under treatment for hypertension, 56 (9%) were under treatment for dyslipidaemia, and 87 (14%) were under treatment for diabetes. However, a significant proportion (24%) of them, despite abnormal metabolic, hormonal, or pressure values, were not taking any treatment. Subjects taking steroid hormones and other drugs known to affect directly testosterone levels were not included in the study (Figure 1).

### 3.2. What Is the Prevalence of Cardiovascular and Metabolic Risk Factors in Unselected Men with ED?

One-third of patients (30.8%) met the diagnosis of metabolic syndrome (according to ATPIII criteria). Specifically, 33.2% had reduced HDL cholesterol, 23.6% had increased waist circumference, 67% had high blood pressure (of which 49.9% had systolic, and diastolic had systolic and diastolic hypertension, 42% had isolated systolic and 8.1% had isolated diastolic hypertension), 37.5% had hypertriglyceridaemia, and 31.7%, had high blood glucose.

**Figure 2:**
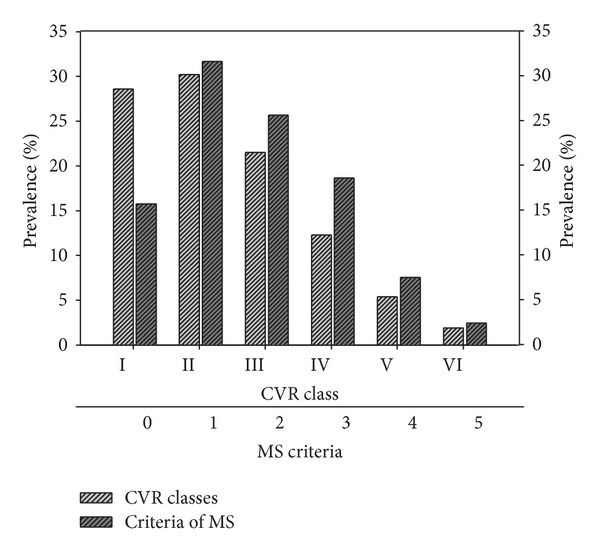
Prevalence of CV risk (CVR) classes and number of metabolic syndrome (MS) criteria in the study population.

**Figure 3:**
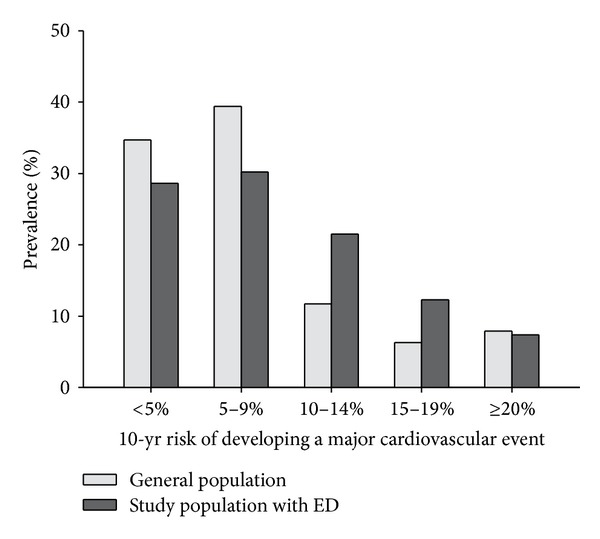
Distribution of CV risk in the general population and in the ED population.

Stratification of CV risk (according to the* Progetto Cuore* risk chart) categorised 28.6% and 30.2% of subjects as classes I and II (10-year risk of incipient MACE <10%), 21.5% as class III (10–15%), 12.3% as class IV (15–20%), and 5.4% and 1.9% as classes V and VI (>20% risk). A comparison of the prevalence of metabolic syndrome components in the CV risk stratification is shown in Figure 2. This analysis shows that while the distributions of metabolic syndrome criteria and CV risk scores were similar in low and high risk classes (I-II or V-VI), for the intermediate classes, they were clearly divergent. A comparison of the CV risk of the ED population compared to the age-matched general population (*n* = 68890, mean age 54.9) is shown in Figure 3. The comparison was possible since the CV risk was assessed using the computerized engine (*Progetto Cuore*) distributed by the NIH; the same algorithm is used for periodical remote monitoring of CV risk by GPs. The 68890 men used in the comparison were age-matched men who were entered in the system during the same time frame of our study. It can be seen that the ED population had a significantly higher prevalence of moderate CV risk (classes III and IV: 18% versus 33.8% *P* < 0.001), an essentially similar prevalence of high CV risk (classes V and VI: 7.9 versus 7.4%) and a lower prevalence of low CVR.

### 3.3. Does the Diagnostic Criteria of Metabolic Syndrome Add Any Significant Contribution to the CV Risk Chart for Prediction of ED?

The age-adjusted association between the investigated variables, CV risk and ED severity is reported in Table 2. The CV risk, stratification risk was strongly associated with impaired EF, even when controlled for age and smoking. Similarly, PP was associated either with increasing CV risk or with declining IIEF-5 score. Interestingly, the number of metabolic syndrome criteria was not correlated with ED severity as measured by IIEF-5. Although there was a correlation between MS criteria and CV risk class, possibly because the latter includes diabetes, no correlation was found between metabolic syndrome and PP. Total testosterone was inversely correlated with age, CVR, PP, and waist circumference, but not with MS. In contrast, waist circumference was directly correlated with CVR, PP, metabolic syndrome, and TT, but not with IIEF-5 score. All correlations remained significant when the analyses were performed on subjects not on antihypertensive medications. After adjustment for confounding factors (age, smoking, and center), no significant correlation was found between metabolic syndrome and other investigated variables.

### 3.4. What Are the Best Determinants of the Severity of ED in Real-Life Settings for Routine Use in Clinical Practice? 

Regression analysis was performed to investigate the best determinants of erectile function measured as total IIEF-5 score.  Table 3 reports the odds ratio for severe ED when the single components of the cardiometabolic risk and hormonal variables were individually tested in a model adjusted for age and centre. In a stepwise model, entering all variables significantly correlated with IIEF-5 score, while CV risk class (as number) was found to be the best independent determinant of IIEF-5 total score (std *β* = −0.279, *P* < 0.001, and Adj *R* ^2^ = 7.4%). When cumulative variables, CV risk class, or number of metabolic syndrome criteria were excluded from the model, the following variables were found to be significant independent contributors to IIEF-5: age (std *β* = −0.199, *P* = 0.001, and Adj *R* ^2^ = 3.6%), PP (std  *β* = −0.144, *P* = 0.0,  and ΔAdj *R* ^2^ = +2.0%), and testosterone (std  *β* = 0.213, *P* = 0.040, and  ΔAdj *R* ^2^ = +1.2%). The role of CVR, age, testosterone, and PP was confirmed when IIEF-5 categories or clinical severity of ED classes were used (data not shown). Among the five components of the metabolic syndrome, only glycaemia was a significant determinant of IIEF-5 score (std *β* = −0.147, *P* = 0.016, and Adj *R* ^2^ = 1.8%). Finally, a comprehensive model of logistic regression analysis for the identification of subjects with severe ED, including global scores (CVR class and MetS diagnosis), was tested. This confirmed that CV risk class was the single best significant contributor to the model, with 88% of affected subjects correctly predicted (OR = 1.57 CI: 1.29–1.83).

### 3.5. Does PP Depend on Androgen Levels?

**Figure 4:**
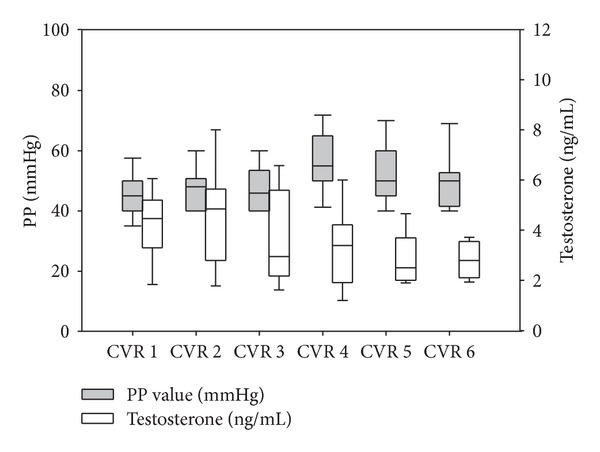
Pulse pressure and serum testosterone stratified according to cardiovascular risk.

**Figure 5:**
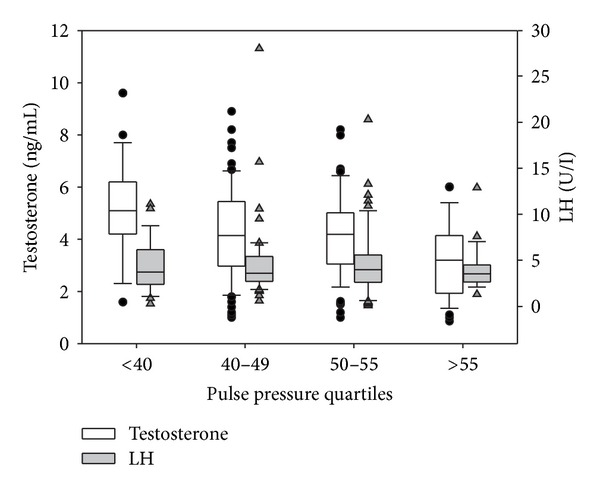
Distribution of testosterone and LH levels stratified according to quartiles of pulse pressure. Open boxes are total testosterone values (left vertical axis) and solid gray boxes are LH values (right vertical axis).

The distribution of PP and testosterone values according to CV risk class is reported in Figure 4. Significantly higher PP values were found in classes above CVR-III (class I versus IV, *P* < 0.001, I versus V, *P* = 0.010, II versus VI, *P* < 0.001, and III versus IV, *P* < 0.001). Testosterone was also stratified for CVR, showing an inverse trend, with higher CV risk associated with lower testosterone levels (class I versus IV, *P* = 0.031, I versus V, *P* = 0.022, and II versus IV, *P* = 0.011). The distribution of testosterone and LH according to PP is reported in Figure 5. Interestingly, the progressive decline in testosterone was not accompanied by a compensatory rise in LH indicating that high CV risk is associated with secondary, rather than primary, hypogonadism. The novelty of this finding is the progressive impairment to the hypothalamo-pituitary-testicular axis with increasing CV risk and PP (Figure 5). The determinants of PP are analysed in Table 4. Age, waist circumference, and testosterone were found to be determinants for PP, explaining up to 12% of variability. BMI, glycaemia, HDL, and triglycerides did not contribute to the model.

### 3.6. Is Response to Vardenafil Maintained in High CV Risk Subjects?

**Figure 6:**
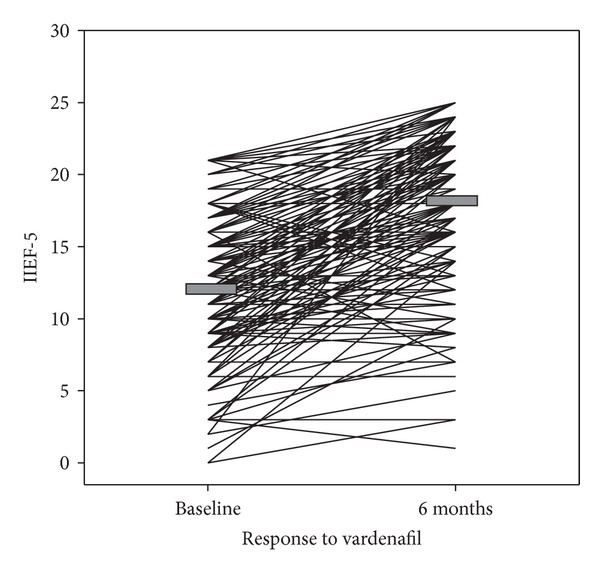
Response to vardenafil measured by the international index of erectile function (IIEF-5).

Treatment with vardenafil determined a significant increase in IIEF-5 scores from baseline (12.45 ± 4.96 versus 18.40 ± 5.09, *P* < 0.001; see Figure 6). At baseline, the IIEF-5 score was <21 for the entire study population, while after treatment it had normalized in 32% of the subjects, with 62% achieving an improvement ≥5 points and 81% showing an improvement variation in the ED severity class (severe, moderate-to-severe, mild-to-moderate, and mild). Consistent results were obtained even in men with moderate and severe ED (40.6%, with IIEF-5 score <12 at baseline). In these men, there was a mean increase in IIEF-5 score of 112% (from 7.58 ± 2.95 to 16.06 ± 5.61, *P* < 0.001). In the remaining patients (59.4%) with IIEF-5 score >12, the mean increase was 27% (from 15.78 ± 2.85 to 20.00 ± 4.00, *P* < 0.001). The global net increase was 48%.

**Figure 7:**
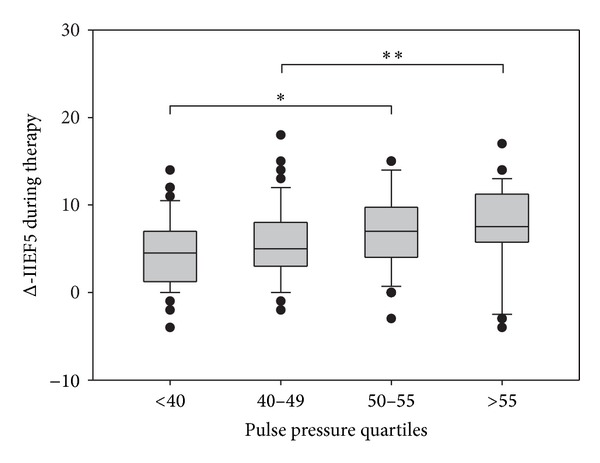
Change in IIEF-5 score in the population stratified according to quartiles of pulse pressure.

**Figure 8:**
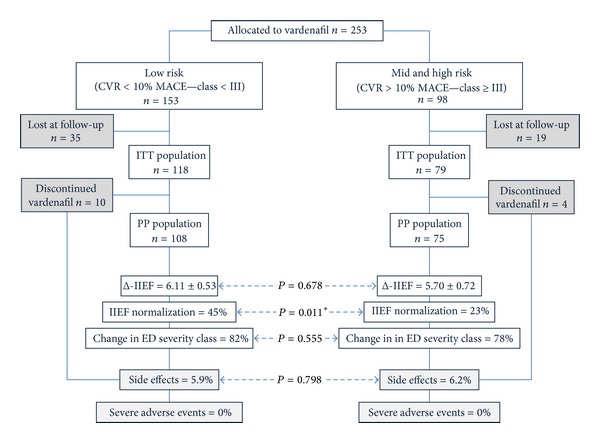
Comparisons of efficacy and safety for vardenafil treatment in subject with low versus high cardiovascular risks.

When stratified for PP, the IIEF-5 score increase tended to be more pronounced in subjects with higher PP quartiles (class 1 versus 3, *P* = 0.031; Figure 7). The efficacy of vardenafil in subjects with low and mid-high CV risk is compared in Figure 8. Vardenafil proved the noninferiority in the primary endpoint of the study; an essentially identical change (increase) in IIEF-5 score was observed for both low and high CVR subjects. As expected, the baseline difference in IIEF-5 score led to a significantly lower percentage of subjects achieving a normal IIEF-5 score in last visit among the high risk men. Nevertheless, there was no difference in the percentage of subjects achieving an improved ED class. Response to vardenafil was equal in men both with and without vascular disease on penile duplex ultrasound (data not shown).

### 3.7. Is Vardenafil Safe in High CV Risk Subjects?

The most common treatment-emergent adverse events, occurring in ≥2% of any treatment group (safety population), were headache (4.1%) and flushing (3.6%). In the six-month treatment period, 54 patients were lost to follow-up or discontinued the medication due to its cost. Overall, 14 discontinued vardenafil (<6% of overall population) because of reported drug-related non-serious adverse events such as headache and facial and neck flushing after the first three doses. No serious adverse event was reported during the study.

A small reduction in diastolic blood pressure (82.76 ± 8.31 versus 80.96 ± 6.79; *P* = 0.043) was observed during treatment. There was also a slight reduction in systolic blood pressure (131.9 ± 15.0 versus 129.1 ± 10.6 mmHg; *P* = 0.058), which however was not statistically significant. There was no significant change in PP (49.30 ± 10.81 versus 48.35 ± 8.83; *P* = NS) following six months of on-demand vardenafil administration. No significant difference was seen in discontinuation rate, side effects, and follow-up loss frequencies between low and mid-high CV risk classes (Pearson's *χ* ^2^ test), indicating that the drug is safe and well tolerated in ED subjects with an elevated cardiovascular risk (Figure 8).

## 4. Discussion

This Italian nationwide observational study on a large cohort of middle-aged men suffering from ED demonstrates that ED severity is closely correlated with increased CV risk. This correlation is the result of the combined effect of diabetes, hypertension, arterial stiffness, and hypogonadism, measured using a validated regional risk score chart developed by the* Progetto Cuore*. The SIAMS-ED survey also revealed, for the first time, that androgens are significant contributors to arterial stiffness of ED patients that exhibit an alteration of the HPG axis. In contrast, we showed that, when CV risk is correctly estimated, the diagnosis of metabolic syndrome does not add any useful information. The major contribution of the diagnostic criteria for metabolic syndrome was found to be increased waist circumference, a causative factor of hypoandrogenism.

This study is among the very few prospective, multicentre studies to offer a real-life estimate of global CV risk and prevalence of metabolic syndrome in ED population. Compared to other Italian surveys [22, 32], we found a higher prevalence of subjects at a moderate risk of MACE, but a substantially similar prevalence in the diagnosis of metabolic syndrome compared to non-ED subjects.

Finally, with the limitation of a nonrandomized, single-arm trial, this real-life setting interventional study showed that vardenafil's response appears durable, preserved independent of the severity of cardiometabolic impairment, and safe even in high CV risk subjects. The latter issues are of particular relevance for practicing physicians in view of the growing interest for long-term, choric use of PDE5i in aging men with cardiovascular disease.

### 4.1. CVR in the ED Population

ED has been identified as an independent risk factor for CVD [9]. In the prostate cancer prevention trial, men with ED were reported to have an approximate two times higher risk of CVD than men without ED [33]. Similarly, in patients seeking treatment for ED, the presence of arteriogenic ED was significantly associated with an increased risk of MACE [34]. Based on these associations, international guidelines recommend in-depth risk factor screening in all subjects with ED prior to symptomatic treatment with PDE5i [35]. However, knowledge of the detection rate of previously unknown underlying medical conditions in men with ED is limited to retrospective, single-centre studies. In the Men's Attitudes to Life Events and Sexuality (MALES) study, 64% of men with ED self-reported one or more underlying condition [36]. In the SIAMS-ED survey, nearly half of the ED population had a previous diagnosis of a vasculogenic or metabolic disorder and 42% were under medical treatment, with figures similar to those of the general population. However, we also diagnosed unknown cardiovascular or metabolic disorders in 24% of our ED subjects (Figures 2 and 3). These numbers are nearly double those reported in a recent UK study [37], where the rate of discovery of previously unknown conditions was 11.53%. The discrepancy is likely due to methodological differences, rather than regional characteristics. The SIAMS-ED study was in fact prospectively designed, while Kirby's study was based on disease registry. Finally, some studies tended to show a decline in the rate of discovery or association of CVD with ED, after PDE5 became widely available [38]. Our data do not support this finding, underlying the largely unmet need of CV risk prevention in ED patients.

### 4.2. PP and Androgens

A relationship between testosterone and arterial stiffness was first suggested by Rosano et al. [39], who found that short-term intravenous testosterone administration reduced time to exercise-induced myocardial ischemia, probably due to a direct coronary artery-relaxing effect. Androgen withdrawal has also been associated with reduced large artery compliance [40]. Longitudinal analysis revealed that the effects of testosterone on arterial stiffness could still be detected, in healthy subjects, when testosterone was measured 5 to 10 years later, supporting a long-term influence [41].

We were the first to document that the association between testosterone and PP had implications for arteriogenic ED [18, 42], as also confirmed by an increased augmentation index measured by peripheral arterial tonometry [43]. In the present study, we showed, for the first time, that the association between PP and T was maintained even when adjusted for waist circumference (Table 4). In addition, we demonstrated that the hypoandrogenism developing in the high CV risk subjects is not accompanied by LH rise, suggesting hypothalamic-pituitary insufficiency (Figure 5) [20, 30, 44]. These associations were maintained even after correction for the use of antihypertensive medications. PP thus seems to be a reliable marker of cardiovascular and sexual health, with higher values associated with altered testosterone.

Our study is also the first to address, whether the association between PP and hypogonadism in ED depends on systolic or diastolic components of BP. The relationship between testosterone levels and hypertension is not completely clear in the available literature. Some studies have found no association [18, 45, 46], neither a negative [47] nor a positive association [48, 49] between systolic blood pressure and androgens. In contrast, the majority of studies found a weaker association with diastolic blood pressure. Our study confirms that the main contributor to elevated PP is systolic hypertension, although diastolic hypertension was more frequent in hypogonadal men. We also showed that the association between PP and androgens was maintained when PP was normalized for diastolic blood pressure values and it was independent of medications known to affect androgen status [50].

### 4.3. Cardiovascular versus Metabolic Diagnostic Categories

There is an open debate on the value of metabolic syndrome diagnosis in various clinical settings [51–53]. In contrast with some previous studies [6], the SIAMS-ED survey seems to show that the CV risk chart score is better than the metabolic syndrome criteria in predicting the severity of ED [7, 54]. This finding is consistent with the recent publication of The emerging risk factors collaboration, who found that, when additional information is available from standard risk score, simple adiposity measures (BMI or waist circumference) provide little or no additional information on CV risk in the general population [55]. This prospective study of 221934 subjects concluded that CV risk scores which omit adiposity measures (e.g., Framingham, SCORE, PROCAM, Reynolds and ISS-Progetto Cuore Engine) are not further improved by including single or combined measures of adiposity. Our survey extends this finding to ED subjects, showing that a good CV risk chart is a better prognostic tool than metabolic syndrome criteria. The major contribution of metabolic impairment, through an increased waist circumference, was that of further reduction of androgen levels [20]. The circle is then closed, considering low testosterone levels to be a novel determinant of increased PP for middle-aged men suffering from ED [20]. Given the recently described association between reduced androgens and cardiovascular abnormalities and mortality [56, 57], all hypertensive patients should be screened for hypogonadism [20].

### 4.4. Response to Vardenafil

The increasing number of risk factors for CVD has been associated with a lower response to conventional treatments [24, 58]. Despite this poor prognosis, we tested vardenafil on demand in middle-aged and older patients who were enrolled independent of their prior use of any phosphodiesterase type 5 inhibitors (PDE5-i) and stratified according to the severity of CVR and found that clinical response was equally detectable regardless of the presence or absence of penile vascular disease (Figure 6).

The efficacy and safety of vardenafil have been tested in several randomized trials, including men with various underlying conditions [25, 59–63]. All these studies documented efficacy that was retained irrespective of the medication used to treat the underlying disorders. However, all these studies were relatively short (12 weeks) and none used a recognized CV risk chart score to stratify at-risk patients. In addition, this is the first time PP quartiles have been assessed as an additional surrogate marker of CVR.

Our study, being non-randomized and single-arm, could not formally assess efficacy; however our data consistently with previous observation [64] confirmed that vardenafil improved erections and was well tolerated in a real-life population of men with cardiovascular impairment, many of whom were also taking concomitant medications. In addition, we showed, in high CV risk classes, normally excluded in sponsored trials, that vardenafil is safe and well tolerated regardless of the use of concomitant antidiabetic medication [65], multiple antihypertensives [60], or lipid-lowering agents [66]. Specifically, we found no difference in response in statin users than in nonusers (Figure 8).

Vardenafil was well tolerated over the 6-month period, with a low incidence of adverse events. This underestimated incidence might be due to the fact that data were only recorded at follow-up visits; other studies have shown that a greater number of serious versus mild adverse events are reported by physicians during postmarketing drug surveillance [67]. In our cohort, the treatment-emergent adverse events were mostly mild-to-moderate headache and flushing, affecting <5% of the treated population. There were no drug-related serious adverse events. Furthermore, SBP, diastolic blood pressure, and heart rate, particularly relevant in the high CV risk group, were not significantly altered by vardenafil treatment (Figure 8). The latter findings are of particular interest given the preliminary data showing that PDE5i could, in the future, be tested as antiremodelling agents [68].

### 4.5. Limitations

This study has several limitations. Regarding the observational study, we acknowledge that PP is not as accurate as other surrogate measures of arterial stiffness, including ultrasonography and tonometry. However, it is an easy, fast, and inexpensive measurement which can be readily obtained in wider populations for epidemiological purposes. A second limitation is that most studies have explored the association between androgen and large arteries, whereas PP or CDU resistance index measures compliance of small peripheral arteries. A third weakness is that only testosterone was recorded and given that SHBG could be significantly altered in diabetic patients; this aspect may have lowered the power of any associations with androgens.

The major limitations of the interventional study are that it was neither randomized nor had a placebocontrolled group (singlearm, open label). However, efficacy over placebo, which is well established, was not our primary. The primary objective was in fact to compare responsiveness in different CV risk classes. As the study included nonnaïve patients with ED, the efficacy and safety may have been somewhat biased by any prior use of another PDE5i. However, prior-PDE5i users were equally distributed among the two groups (low and high CV risk groups). In any case, we believe that this inclusion criteria would have reduced, rather than increased, the response rate and therefore should have not altered the direction of the observed trends. Finally, only one posttreatment evaluation was available for efficacy assessment, compared to the monthly visits performed in many trials. However, this is much closer to follow-up times in a real-life public outpatients clinics. Furthermore, our study is among the few exploring the effects after six months of treatment, as the vast majority terminate their observations after 3 months. Once again, this design, if carrying any relevant effect, would have been in the direction of lowering the response rate. The efficacy of vardenafil in difficult-to-treat populations would thus seem to be indirectly confirmed.

## 5. Conclusions

The SIAMS-DE study demonstrates for the first time in large multicentre independent prospective analysis that PP is related to many aspects of cardiometabolic compensation, such as BMI, waist circumference, and testosterone levels, which are in turn related to a higher incidence of MACE in the general and ED population. We found that a progressive defect in hypotalamo-pituitary-gonadal feedback is associated with increased CV risk and contributes to increased cardiovascular stiffness through an association with systolic, but not diastolic, BP. This study gives another insight into the fact that the presence and severity of ED should be considered a sentinel symptom of cardiometabolic involvement and should merit hormonal assessment and tough intervention [20, 23]. This study demonstrates that concomitant antihypertensive treatment does not affect the efficacy of vardenafil and that the improvement in IIEF-5 is preserved even in higher CV risk men. This aspect is very new, as PDE5-i are usually found to lose efficacy in the presence of severe concomitant conditions like hypertension, diabetes, dyslipidaemia, or CAD. In conclusion, we demonstrated that vardenafil is a valuable option for the treatment of ED even in high-risk, difficult-to-treat populations.

## Figures and Tables

**Table 1:** Baseline parameters (604 recruited patients). Data are presented as mean ± standard deviation when normally distributed, median [25th–75th percentiles] when skewed, and as percentages when categorical.

Parameter		
Age	Years	55.3 ± 12.08
Anthropometrics	Weight (kg)	82.9 ± 13.46
	BMI (kg/m^2^)	27.4 ± 4.15
	Waist circumference (cm)	99.1 ± 11.84
Metabolism	Glycaemia (mg/dL)	102 [94–114]
	HDL cholesterol (mg/dL)	45.9 ± 11.03
	Triglycerides (mg/dL)	139 ± [100–175]
Cardiovascular	Systolic BP (mmHg)	131.41 ± 10.48
	Diastolic BP (mmHg)	82.17 ± 6.96
	Pulse pressure	49.25 ± 10.4
	Heart rate	75.88 ± 8.17
Hormonal	FSH (IU/mL)	6.4 ± 10.3
	LH (IU/mL)	4.86 ± 5.43
	Total testosterone (ng/dL)	4.04 ± 1.7
Penile CDU	Peak systolic velocity (cm/s^2^)	40.4 ± 13.1
	Penile resistive index	0.83 ± 0.27

Prevalence of diagnostic criteria		(%)

CV risk class	I (MACE risk < 5%)	28.6
	II (MACE risk 5–10%)	30.2
	III (MACE risk 10–15%)	21.5
	IV (MACE risk 15–20%)	12.3
	V (MACE risk 20–30%)	5.4
	VI (MACE risk > 30%)	1.9
Metabolic syndrome (yes)	≥3 criteria	30.8
Blood pressure	≥130/85 or treatment	67.0
Triglycerides	≥150 mg/dL (≥1.7 mmol/L)	37.5
HDL cholesterol	≤40 mg/dL (≤1.03 mmol/L)	33.2
Fasting glucose	≥110 mg/dL (≥6.1 mmol/L)	31.7
Waist	≥102 cm	23.6
ED severity (IIEF-5 class)	Mild (17–21)	22.9
	Mild to moderate (12–16)	37.7
	Moderate (8–11)	21.5
	Severe (<8)	17.8

**Table 2:** Partial correlations adjusted for age and smoking.

Variable	IIEF5 score	IIEF5 4 categories	IIEF5 2 categories	CV risk categories	Pulse pressure	MS yes versus no	MS number of criteria	Testosterone
IIEF-5 4 categories	−0.949* *P* = 0.000							
IIEF-5 2 categories	−0.815* *P* = 0.000	0.874* *P* = 0.000						
CVR categories	−0.113^§^ *P* = 0.045	0.156^‡^ *P* = 0.005	0.142^§^ *P* = 0.043					
Pulse pressure	−0.124^§^ *P* = 0.013	0.112^§^ *P* = 0.017	0.111^§^ *P* = 0.018	0.148^‡^ *P* = 0.006				
MS yes versus no	0.093 *P* = 0.305	−0.052 *P* = 0.535	−0.056 *P* = 0.504	0.006 *P* = 0.952	0.081 *P* = 0.315			
MS number of criteria	0.039 *P* = 0.624	−0.026 *P* = 0.726	−0.05 *P* = 0.497	0.093 *P* = 0.267	0.052 *P* = 0.463	0.844* *P* = 0.000		
Testosterone	−0.031 *P* = 0.726	−0.078 *P* = 0.274	−0.017 *P* = 0.811	−0.149^§^ *P* = 0.046	−0.057 *P* = 0.453	−0.072 *P* = 0.566	−0.123 *P* = 0.253	
Waist circumference	−0.003 *P* = 0.962	0.021 *P* = 0.743	−0.048 *P* = 0.453	0.175^§^ *P* = 0.012	0.146^§^ *P* = 0.018	0.302^‡^ *P* = 0.003	0.245^‡^ *P* = 0.008	−0.197^§^ *P* = 0.035

IIEF-5 categories: mild ED versus mild to moderate ED versus moderate ED versus severe ED.

IIEF-5 2 categories: mild and mild to moderate ED versus moderate and severe ED.

*P < 0.0001, ^†^ *P* < 0.001, ^‡^ *P* < 0.01, and ^§^ *P* < 0.05.

**Table 3:** Odds ratio for severe ED. Variables are individually tested in a model adjusted for age, smoking, and centre. Significant ORs are highlighted in bold.

	Odds ratio	CI	*P* value
BMI	1.037	.980–1.098	.202
Waist (cm)	1.005	.982–1.028	.665
Diastolic BP (mmHg)	.980	.948–1.012	.216
Diastolic hypertension (categorical)	1.192	.727–1.955	.487
Systolic BP (mmHg)	1.000	.982–1.018	.995
Systolic BP (categorical)	1.132	.681–1.883	.633
Pulse pressure (PP)	1.012	.988–1.037	.334
PP normalized for DBP	**5.128**	**1.087**–**24.545**	**.040**
Total cholesterol	.993	.985–1.000	.051
HDL cholesterol	.994	.968–1.021	.677
Triglycerides	1.000	.997–1.003	.951
Fasting glycemia	1.004	.997–1.011	.237
Hb1Ac	**1.296**	**1.027**–**1.634**	**.029**
Diabetes (categorical)	**2.072**	**1.172**–**3.666**	**.012**
Testosterone	**.786**	**.625**–**.988**	**.039**
CV risk class	**1.437**	**1.105**–**1.868**	**.007**

**Table 4:** Linear regression analysis for the determinants of pulse pressure in the study population.

	*B*-coefficient	SE of *B*	*P* value
Variable entered in the model			
(constant)	21.350	12.742	0.098
Age	0.192	0.097	0.041
Total testosterone	−1.231	0.616	0.040
Waist circumference	0.306	0.156	0.044
Excluded variables			
HDL cholesterol	0.199	0.142	0.166
Triglycerides	0.004	0.011	0.709
Glycaemia	−0.015	0.029	0.613
BMI	−0.622	0.394	0.119

Adj *R* ^2^ = 0.124.
